# Role of AT‐rich interaction domain 1A in gastric cancer immunotherapy: Preclinical and clinical perspectives

**DOI:** 10.1111/jcmm.18063

**Published:** 2023-12-02

**Authors:** Xuemei Zhang, Youzhi Zhang, Qiaoyun Zhang, Mengyao Lu, Yuan Chen, Xiaoyu Zhang, Peng Zhang

**Affiliations:** ^1^ Department of Oncology, Tongji Hospital, Tongji Medical College Huazhong University of Science and Technology Wuhan China; ^2^ School of Pharmacy Hubei University of Science and Technology Xianning China; ^3^ Division of Gastrointestinal Surgery, Department of General Surgery, Huai'an Second People's Hospital the Affiliated Huai'an Hospital of Xuzhou Medical University Huaian China

**Keywords:** ARID1A, gastric cancer, genome instability, immune microenvironment, immunotherapy

## Abstract

The application of immune checkpoint inhibitor (ICI) using monoclonal antibodies has brought about a profound transformation in the clinical outcomes for patients grappling with advanced gastric cancer (GC). Nonetheless, despite these achievements, the quest for effective functional biomarkers for ICI therapy remains constrained. Recent research endeavours have shed light on the critical involvement of modified epigenetic regulators in the pathogenesis of gastric tumorigenesis, thus providing a glimpse into potential biomarkers. Among these regulatory factors, AT‐rich interaction domain 1A (ARID1A), a pivotal constituent of the switch/sucrose non‐fermentable (SWI/SNF) complex, has emerged as a promising candidate. Investigations have unveiled the pivotal role of ARID1A in bridging the gap between genome instability and the reconfiguration of the tumour immune microenvironment, culminating in an enhanced response to ICI within the landscape of gastric cancer treatment. This all‐encompassing review aims to dissect the potential of ARID1A as a valuable biomarker for immunotherapeutic approaches in gastric cancer, drawing from insights garnered from both preclinical experimentation and clinical observations.

## 1 INTRODUCTION

Gastric cancer (GC) is a widespread type of cancer and ranks as the third leading cause of cancer‐related deaths worldwide. More than 70% of new cases and deaths from GC occur in developing countries. Two main classification systems are commonly used to categorize GC. The first one divides it based on where the tumour is located, classifying it as cardia or non‐cardia. The second system, called Lauren's classification, sorts GC into three main groups: diffuse, intestinal and mixed types.^1^, ^2^ GC is linked to several risk factors, which encompass *Helicobacter pylori* infection, tobacco smoking, a diet rich in meat, obesity and alcohol consumption. Additionally, a person's genetic makeup and socioeconomic status can also influence their susceptibility to GC.^3^ GC is frequently diagnosed at advanced stages, leading to poor prognoses, primarily because specific clinical symptoms for early detection are lacking.^4^, ^5^ In cases of advanced GC (AGC), systemic chemotherapy remains the primary treatment option. However, the median overall survival (OS) for AGC patients undergoing conventional chemotherapy is only approximately 12 months.^6^ Therefore, we urgently require advanced diagnostic techniques and new drugs to thoroughly study molecular features and identify possible new treatment targets for people with GC.^7^, ^8^, ^9^

In recent times, ICI therapy employing monoclonal antibodies has emerged as a promising approach for treating GC. Immune checkpoints, including cytotoxic T lymphocyte antigen 4 (CTLA‐4), programmed cell death protein 1 (PD‐1) and its ligand programmed cell death ligand 1 (PD‐L1), serve as crucial elements in suppressing T‐cell‐mediated immune responses. They play a pivotal role in enabling cancer cells to evade immune surveillance. ICI are monoclonal antibodies designed to target these immune checkpoints. By reducing the signals that block T‐cell activation, ICI help T cells overcome control mechanisms, resulting in strong and effective responses against tumours.^10^, ^11^ Tumours expressing PD‐L1 have been associated with unfavourable outcomes in GC patients.^12^ Yet, this particular group of patients shows promise for treatments with anti‐PD‐L1 antibodies like atezolizumab, durvalumab and avelumab, as well as anti‐PD1 antibodies such as nivolumab, pembrolizumab, sintilimab, tislelizumab and retifanlimab. Another potential target for immune checkpoint inhibition is CTLA‐4, and antibodies like ipilimumab can be used for this purpose. CTLA‐4 is a receptor found on T cells, and when it binds, it has the ability to reduce T‐cell activation. CTLA‐4 exerts its inhibitory effects both intrinsically, through phosphatase recruitment and inhibition of transcription factors, and extrinsically, by competing with CD28 for binding to costimulatory CD80/86 ligands.^11^ Furthermore, CTLA‐4 is expressed by regulatory T cells (Tregs), and therapeutic anti‐CTLA‐4 antibodies may also enhance anti‐tumour immunity by depleting Tregs and neutralizing their immunosuppressive functions. A growing amount of evidence suggests that when you combine ICIs with other treatments or use two ICIs together, it can greatly improve the treatment's effectiveness while still being well‐tolerated. Additionally, using predictive biomarkers helps us accurately identify patients who are likely to have a positive response to ICIs. As a result, the two key challenges in ICI therapy involve the screening of appropriate combinations to enhance efficacy and the identification of dependable biomarkers for predicting patient responses to ICIs.^13^, ^14^

Gastric cancer is a complex process marked by the gradual accumulation of various genetic and epigenetic changes. These alterations ultimately lead to the activation of oncogenes and the inactivation of tumour suppressor genes. Among these changes, genetic alterations are particularly important. For example, mutations in genes like p53, KRAS, PIK3CA, ARID1A, MLL3, MLL and PIK3CA, along with increases in C‐MET, ERBB4 and CD44, are often seen in gastric cancer. These genetic changes play a crucial role in the formation of GC.^15^ The emerging body of evidence emphasizes the substantial role played by perturbed epigenetic mechanisms in driving the progression of gastric tumorigenesis.^16^ This comprehensive review aims to shed light on how ARID1A's inadequacy affects genome stability, its role in shaping the tumour's immune environment and explore potential interactions with ICI therapy in the context of GC.

## 2 EMERGING ROLE OF ARID1A IN GC IMMUNOTHERAPY

### 2.1 Overview and canonical function of ARID1A

AT‐rich interaction domain 1A, also known as BAF250a or SMARCF1, is a component of the SWI/SNF chromatin remodelling complex and is commonly mutated in many types of cancer. In GC, ARID1A mutations are observed in 8% to 33% of cases and are linked to worse survival outcomes for patients.^17^, ^18^, ^19^, ^20^, ^21^, ^22^ SWI/SNF complexes employ ATP hydrolysis to disrupt DNA‐histone interactions, thereby facilitating the movement of nucleosomes and modulating DNA accessibility to transcriptional and co‐regulatory machinery. The ARID1A gene is situated on chromosome 1p36.11 and comprises 20 exons that encode a 240 kDa protein predominantly located in the cell nucleus.^20^ ARID1A possesses two functional domains: an N‐terminal DNA‐binding domain known as AT‐rich binding domain (ARID), which spans approximately 100 amino acids, and a C‐terminal functional domain housing three leucine‐rich sequences for glucocorticoid receptor binding.^23^

AT‐rich interaction domain 1A, along with its homologous protein ARID1B, exhibits a mutually exclusive association with several other proteins, including the ATPase subunits SMARCA2 (BRM) and SMARCA4 (BRG1). Together, they form the BRG1‐associated factor (BAF) SWI/SNF chromatin remodelling complexes.^24^, ^25^ It's noteworthy that, akin to ARID1A, several subunits of the SWI/SNF complex have multiple paralogs. Some of these paralogous subunits are also mutually exclusive, and the presence of variant subunits can contribute to the specificity of target selection. This combinatorial assembly strategy is believed to underlie the target and lineage specificity observed in different forms of SWI/SNF complexes.^26^, ^27^, ^28^ ARID1A is believed to be involved in bringing BAF complexes to specific DNA regulatory sites by interacting with other transcriptional factors or cofactors. Earlier research has shown that ARID1A can interact with nuclear hormone receptors and P53, particularly when these factors are attached to their signalling molecules (ligands). This interaction boosts the activity of these transcription factors.^29^, ^30^ Conversely, the ARID domain of mammalian ARID1A displays general DNA‐binding properties without specific sequence restrictions, potentially facilitating the improved affinity of BAF complexes to chromatin for specific targets in vivo.^31^

The stability of ARID1A depends on where it is located within the cell. Nuclear ARID1A is generally less stable compared to its cytoplasmic form. This is mainly because it is quickly broken down by the ubiquitin‐proteasome system.^32^ Mutations in the ARID1A gene, which are typically nonsense or frameshift mutations, result in the loss of protein expression.^33^ When in‐frame deletions affect the agreed‐upon nuclear export signal, the ARID1A protein's levels decrease. This happens because it gets stuck in the nucleus and is later broken down. These basic biological processes control where ARID1A is located within the cell and how stable it is. Additionally, when subjected to DNA damage signals in gastric cells, ARID1A is known to be targeted for degradation by ubiquitin E3 ligase complexes.^34^, ^35^ A recent study has identified the E3 ligase TRIM32 as a component of the ubiquitin‐proteasome system that promotes squamous cell carcinoma by facilitating the degradation of ARID1A protein. Conversely, the deubiquitinase USP11 has been shown to inhibit squamous cell carcinoma by stabilizing ARID1A protein through its interaction with syndecan‐2 (SDC2). This suggests the existence of a regulatory axis, involving TRIM32, USP11 and SDC2, which plays a pivotal role in determining the stability of ARID1A and, consequently, its impact as an oncogene or tumour suppressor.^36^

A common characteristic of non‐coding RNAs (ncRNAs) is that they are transcribed from the genome but do not serve as templates for protein synthesis,^37^ thus exerting their biological functions at the RNA level (as summarized in Table 1).^47^ In recent years, microRNAs (miRNAs) have emerged as key players in the development of gastrointestinal tumours, with regulatory roles impacting the proliferation, migration and invasion of tumour cells. These regulatory activities can have either oncogenic or tumour‐suppressive effects^48^ For instance, Yang et al.^49^ reported elevated levels of miR‐7641 in gastric cancer tissues compared to normal para‐cancer tissues. Their study revealed that ARID1A is a gene targeted by miR‐7641, and in gastric cancer tissues, high levels of miR‐7641 are linked to low ARID1A expression. This indicates that miR‐7641 directly regulates ARID1A in a negative way in gastric cancer.^50^ In a separate study, it was found that when miR‐223‐3‐p is overexpressed, it significantly boosts the growth, movement, and invasion of gastric cancer cells while decreasing cell death (apoptosis). Conversely, when miR‐223‐3‐p is reduced, these activities decrease significantly. Notably, ARID1A can effectively counteract the proliferation and invasion of gastric cancer cells that have an overexpression of miR‐223‐3‐p. These findings suggest that miR‐223‐3‐p behaves as an oncogene in gastric cancer by targeting ARID1A.^51^ ARID1A also exhibits tumour‐suppressive functions in hepatocellular carcinoma (HCC) by inhibiting interactions with the long non‐coding RNA (lncRNA) MVIH through the ARID domain or the C‐terminal ARID1A protein‐binding domain.^52^ Furthermore, in breast cancer, ARID1A has been reported to inhibit the expression of another long non‐coding RNA (lncRNA) called UCA1. This occurs by ARID1A's regulation of chromatin accessibility for the transcription factor CEBPα. To sum up, non‐coding RNAs, such as miRNAs and lncRNAs, play crucial roles in controlling various aspects of tumour development and progression. They can function as either oncogenes or tumour suppressors by influencing the expression and actions of genes like ARID1A. This influence impacts critical processes in cancer, including cell growth, movement, invasion and cell death. Understanding these complex regulatory networks is essential for advancing our understanding of cancer biology and identifying potential targets for therapy.

**TABLE 1:** microRNA (miRNA) associated with AT‐rich interaction domain 1A (ARID1A) in gastric cancer.

miRNA	Role	Target genes or pathways	Effects of up‐regulated ARID1A on miRNA	Reference
miR‐221	Oncogene	ARID1A	Inhibit	[38]
miR‐376c	Oncogene	KLF15	Inhibit	[39]
miR‐125a‐5p	Oncogene	PI3K/AKT	Inhibit	[40]
miR‐223‐3p	Oncogene	ARID1A	Inhibit	[41]
miR‐21	Oncogene	P53/mTOR signalling pathway	Inhibit	[42]
miR‐7641	Oncogene	ARID1A	Inhibit	[43]
miR‐181b	Oncogene	SMAD7/TGF‐β signalling pathway	Inhibit	[44]
miR‐370	Oncogene	AKT signalling pathway	Inhibit	[45]
miR‐148a	Tumour suppressor	DNMT1	Promote	[46]
miR‐335	Tumour suppressor	Bcl‐2	Promote	[115]
miR‐200b	Tumour suppressor	ZEB2	Promote	[116]
miR‐409‐3p	Tumour suppressor	PHF10/C‐myc	Promote	[117]
miR‐182‐5p	Oncogene	mTOR signalling pathway	Inhibit	[118]
miR‐429	Tumour suppressor	C‐myc	Promote	[119]

### 2.2 Effects of ARID1A on genome stability

DNA mismatch repair (MMR) is a crucial mechanism for maintaining genome stability, primarily by correcting base‐to‐base and small insertion/deletion mispairing errors that occur during DNA replication. In GC, defects in MMR are common and can result from mutations in MMR genes or the hypermethylation of their promoters.^53^ MMR deficiency leads to a condition known as microsatellite instability (MSI), characterized by the loss of MMR function and an exceptionally high rate of sequence mutations in tumour cells.^54^ One widely accepted theory posits that ARID1A acts as a ‘caretaker’ gene, functioning as a tumour suppressor by preventing sequence mutations and chromosomal structural abnormalities.^55^ ARID1A is thought to be involved in various processes, including DNA damage repair, mismatch repair, DNA decatenation and controlling DNA accessibility. These functions collectively contribute to its role in preserving genome stability and preventing the development of tumours.

AT‐rich interaction domain 1A mutations have been detected in GC cases characterized by MSI. Notably, the loss of ARID1A protein expression is associated with the absence of MMR proteins and MSI in gastric carcinoma. In a previous study involving 489 consecutive primary gastric adenocarcinomas, abnormal ARID1A expression (reduction or loss) was observed in 109 cases and frequent MMR deficiency was reported.^56^ Exome sequencing of GC cases revealed ARID1A alterations in 83% of MSI cases and 73% of cases with Epstein–Barr virus (EBV) infections. These percentages are significantly higher than those observed in non‐EBV‐infected and microsatellite‐stable cases, where ARID1A alterations were present in only 11%.^19^ Another study, encompassing 857 gastric carcinoma cases, including 67 EBV‐positive and 136 MLH1‐depleted cases (corresponding to the MSI‐high phenotype), identified frequent loss of ARID1A expression in EBV‐positive (34%) and MLH1‐deleted (29%) gastric carcinomas. In contrast, the ratio of ARID1A loss in EBV‐negative, MLH1‐preserved gastric carcinoma cases was notably lower.^57^ These discoveries indicate that the lack of ARID1A encourages tumour advancement in GC cells with the MSI‐H phenotype. However, the precise cause‐and‐effect connection between ARID1A mutations and MMR defects is not fully understood. Most ARID1A mutations in MSI GC involve short mononucleotide repeat insertions or deletions, which are uncommon in cases of microsatellite‐stable cancer. This difference may be influenced by the tissue where the tumours originate and other complex factors.

Genome instability can arise from various defects in DNA damage repair (DDR) pathways, with at least five major repair mechanisms identified: homologous recombination (HR), MMR, base excision repair, nucleotide excision repair and non‐homologous end‐joining. Given the well‐established link between specific DDR dysfunctions and carcinogenesis,^58^ ARID1A has been shown to enhance DDR by promoting HR, MMR, and non‐homologous end‐joining.^59^ Notably, mutations in HR DNA damage repair genes have been associated with a high tumour mutational burden (TMB) and a favourable clinical response to checkpoint blockade. In colorectal cancer, HR repair is significantly reduced in ARID1A‐mutant cell lines and fibroblasts compared to their wild‐type counterparts.^60^ Similarly, gastrointestinal (GI) cancers with ARID1A mutations exhibit a notably higher TMB than ARID1A wild‐type GI cancers.^61^ A comprehensive study involving 17,486 cases of tubular GI carcinoma revealed a correlation between specific DDR defects, including ARID1A mutations, and a high TMB in more than 20% of cases. Furthermore, additional research conducted by Li and colleagues on three GI cancer genomics datasets uncovered that ARID1A‐mutated GI cancers with elevated immune activity were associated with higher TMB and lower levels of tumour aneuploidy.^62^ These findings suggest a complex interplay between ARID1A, DDR mechanisms and immune responses in GI cancers.

On a molecular level, ARID1A engages in interactions with crucial components of the DNA damage response pathway. It interacts with the upstream DNA damage checkpoint kinase ATR, which is recruited to DNA double‐strand breaks (DSB) and contributes to the maintenance of DNA damage signals.^63^ Additionally, ARID1A plays a role in recruiting the mismatch repair protein MSH2 to chromatin, thereby facilitating the process of MMR. This interaction occurs through the C‐terminal region of ARID1A and the N‐terminal region of MSH2, and it influences MSH2's function independently of ARID1A's transcriptional regulatory activity.^64^ Furthermore, two independent studies have reported that DNA damage triggers the ubiquitination and degradation of ARID1A via ubiquitin E3 ligases in GC cells.^34^, ^35^ Notably, the TMB in ARID1A mutant GI cancers was found to be significantly higher than in ARID1A wild‐type GI cancers.^61^ Loss of ARID1A expression was observed in 22.5% of gastric adenocarcinomas in two separate patient cohorts and was positively correlated with the loss of expression of MMR proteins.^65^ These findings highlight the multifaceted role of ARID1A in DNA damage response and repair mechanisms in GC.

AT‐rich interaction domain 1A is vital for preserving genome stability as it plays a part in different DNA damage repair processes. An important interaction it has is with topoisomerase IIα (TOP2A), an enzyme responsible for unravelling newly duplicated sister chromatids and ensuring correct chromosome distribution during cell division (mitosis). This interaction is crucial for TOP2A's attachment to chromatin and depends on the ATPase activity of SWI/SNF complexes.^32^ When ARID1A is knocked down, there is an increased formation of anaphase bridges during mitosis, a phenomenon commonly associated with chromosomal instability resulting from TOP2A inhibition. SWI/SNF complexes have also been shown to enhance DNA damage repair. They are recruited to DNA DSB through interactions with acetylated H3 in nucleosomes containing gamma‐H2AX, thereby promoting ATM‐mediated phosphorylation of H2AX around the DSB site. This creates a positive feedback loop that supports efficient DSB repair.^47^ A malfunction in the SWI/SNF complex can potentially undermine DNA damage repair, which may lead to genomic instability. Furthermore, ARID1A's role in responding to DNA damage also involves its interaction with a nonchromatin substrate called Chk2. When ARID1A is lost, it results in an increase in Chk2 levels by affecting the autoubiquitination of the E3 ligase RNF8. As a result, there is a reduction in RNF8‐mediated Chk2 degradation. Inhibiting the ATM/Chk2 DNA damage checkpoint pathway triggers replication stress and the accumulation of cytosolic DNA, activating the innate immune response mediated by the DNA sensor STING in ARID1A‐deficient tumours.^62^ ARID1A is also associated with other DNA damage repair mechanisms, such as non‐homologous end‐joining. It recruits proteins like 53BP1 and RIF1 to facilitate DNA repair through non‐homologous end‐joining mechanisms.^66^ These findings underscore the multifaceted involvement of ARID1A in safeguarding genome stability and responding to DNA damage in GC.

### 2.3 ARID1A and DNA methylation modification

DNA methylation is a crucial epigenetic modification implicated in the development of cancer as it can lead to gene silencing. This process plays a pivotal role in regulating chromatin structure and gene expression.^67^ DNA methylation is a process where a methyl group is added to the fifth carbon position of cytosine. This reaction is carried out by DNA methyltransferases (DNMTs), including DNA methyltransferase 1 (DNMT1), DNMT3a and DNMT3b. Various factors, such as Helicobacter pylori infection, ageing, diet, chronic inflammation and microbial infections, have been recognized as causes of abnormal DNA methylation. These factors worsen the development of GC by causing abnormal methylation in important genes, leading to the suppression of tumour suppressor genes.^68^ DNA methylation serves as a critical mechanism of epigenetic regulation, and hypermethylation of the ARID1A promoter region leads to the silencing of this gene. This epigenetic alteration can have significant implications for GC development and progression.

Epstein–Barr virus‐positive gastric cancer frequently shows mutations in ARID1A and PIK3CA and has a more distinct CpG methylation profile when compared to MSI‐type GC. Moreover, EBV‐positive stomach cancer is known for the amplification of the 9p24.1 locus, which leads to the overexpression of immune checkpoint proteins PD‐L1 and PD‐L2.^69^ Promoter hypermethylation has been linked to decreased ARID1A mRNA expression in various cancer types, including invasive breast cancers and cholangiocarcinoma.^70^, ^71^ Reactive oxygen species have been shown to induce increased methylation of the ARID1A promoter through the action of DNMT1, resulting in the suppression of ARID1A expression in conditions like endometriosis.^72^ Conversely, treatment with alpha‐oxoglutarate has been found to promote the hypomethylation of ARID1A and elevate its expression. This effect is mediated by 10–11 translocation hydroxylase in normal bladder epithelial cells.^73^ A recent study has reported that promoter hypermethylation plays a role in reducing ARID1A expression, disrupting transcriptional homeostasis and contributing to tumour progression in squamous cell carcinoma.^74^ These findings underscore the complex regulatory mechanisms involving ARID1A and DNA methylation in various cancer contexts.

### 2.4 ARID1A mutations shape the tumour immune microenvironment and phenotype

#### 2.4.1 PD‐L1 expression

**FIGURE 1:**
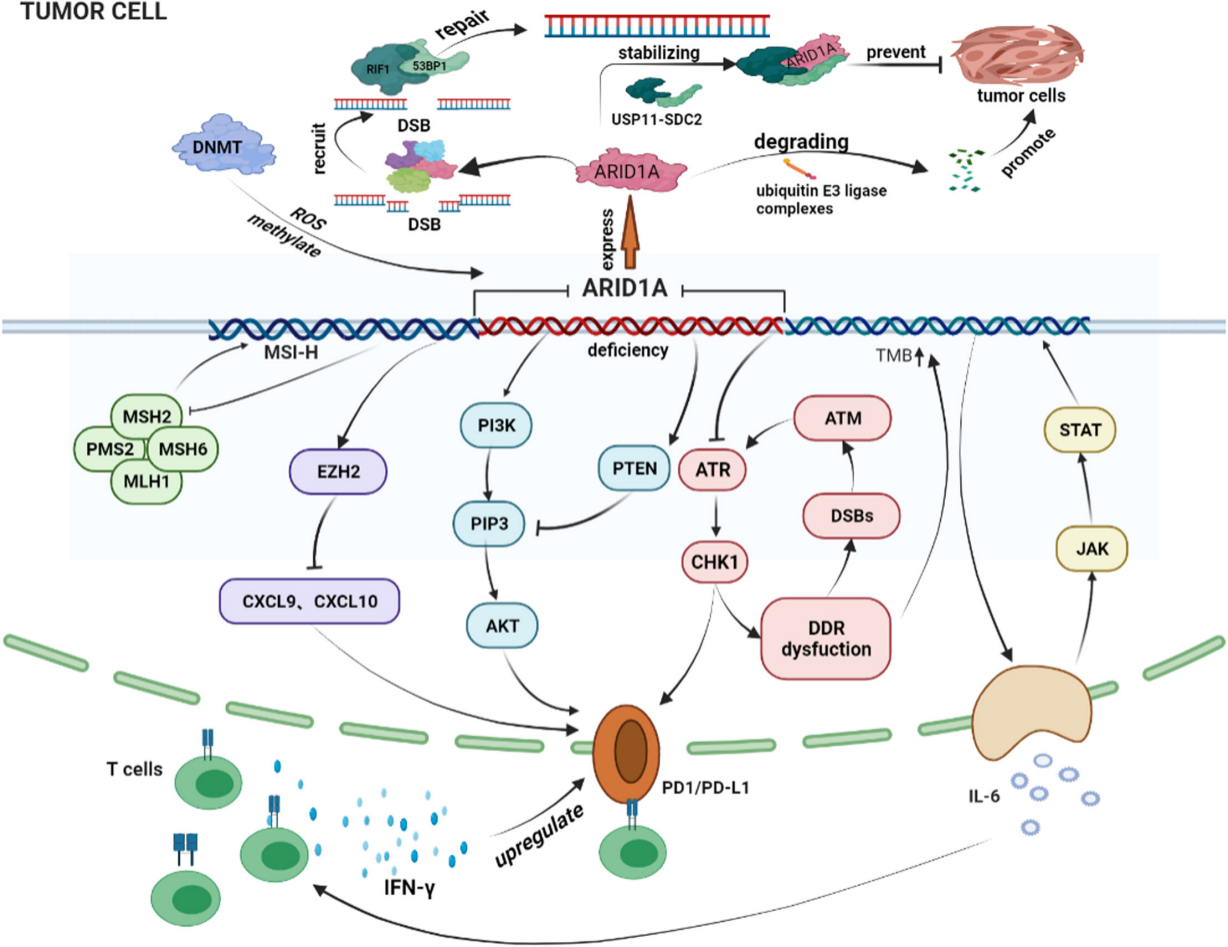
AT‐rich interaction domain 1A (ARID1A) proteins balance the stability and carcinogenic/inhibitory effects of ARID1A through the TRIM32/USP11‐SDC2 signalling pathway. When DNA DSB, ARID1A will recruit other repair proteins to chromatin to regulate mismatch repair (MMR) function and repair DNA. *ARID1A* deficiency induces an increase in expression of PD‐1/PD‐L1 and tumour mutational burden, damage to MMR and regulation of the tumour immune microenvironment.

Programmed cell death ligand 1, the ligand for PD‐1, is expressed in both normal and malignant tissues.^75^ When exposed to interferon‐gamma (IFN‐γ) released by locally activated T cells, tumour cells often upregulate PD‐L1 expression. This increased PD‐L1 expression in tumour cells plays a critical role in promoting resistance to T cell‐mediated immunity, particularly within the tumour microenvironment (TME).^76^ Over time, T cells infiltrating the TME may become exhausted due to prolonged exposure to tumour antigens. This exhaustion is characterized by high expression of PD‐1 and reduced anti‐tumour functionality. Meta‐analysis data have suggested a correlation between PD‐L1 expression and OS in GC.^77^ Furthermore, the deficiency of ARID1A has been associated with an elevation in PD‐L1 expression and alterations in the immune TME. These findings support the idea that loss of ARID1A could potentially serve as a predictive biomarker for the response to ICI therapy, as illustrated in Figure 1.^78^

Kim et al.^79^ have proposed a link between ARID1A and PD‐L1 expression in GC. They conducted experiments on a large cohort and found high PD‐L1 expression in ARID1A‐deficient tumours. To validate these findings, whole tissue sections were used, further confirming the strong association between ARID1A loss and PD‐L1 overexpression. Similarly, another study by Li et al.^68^ reported that gastrointestinal cancer cases with mutant ARID1A were more likely to exhibit high PD‐L1 expression compared to cases with wild‐type ARID1A. These results were consistent with findings from a separate study in which increased PD‐L1 expression was observed in tumours formed in mice with ARID1A‐deficient ovarian cancer cells.^64^ Several mechanisms have been proposed to explain this potential correlation: (1) ARID1A deficiency may increase PD‐L1 expression in tumour cells by activating the phosphatidylinositol 3 kinase/AKT/mammalian target of rapamycin (PI3K/AKT/mTOR) pathway in GC.^79^ (2) ARID1A deficiency might promote DNA DSB, leading to the upregulation of PD‐L1 in tumour cells through the ATM/ATR/Chk1 signalling pathway.^80^ (3) The gene Cd274, which encodes PD‐L1, is considered a direct target of ARID1A.^81^ These mechanisms suggest a complex interplay between ARID1A and PD‐L1 regulation in cancer cells.

#### 2.4.2 Infiltrating immune cell function

The immune system plays a crucial role in both cancer development and the body's ability to fight tumours. Activation of the immune system during tumour growth can be beneficial, leading to tumour rejection. Immune cells in the tumour microenvironment (TME), especially lymphocytes and macrophages, have a significant impact on tumour progression. Immune cells like natural killer cells, CD8+ T cells and type 1 helper CD4+ T (Th1) cells have strong anti‐cancer activity and are often linked to positive outcomes in human tumours. On the other hand, regulatory T cells (Treg cells) can suppress the functions of CD4+ and CD8+ T cells, contributing to immune suppression in cancer. Macrophages and monocytes in the TME can be categorized as M1 (pro‐inflammatory) or M2 (alternatively activated) types, with tumour‐associated macrophages frequently showing an M2‐like phenotype. The presence of immune cells infiltrating the tumour is closely connected to the prognosis of various cancers, including GC.^82^ Inactivation of ARID1A is known to impair T‐cell infiltration into tumours and reduce anti‐tumour immunity.^83^ However, analysis of GC data from The Cancer Genome Atlas (TCGA) revealed that loss of ARID1A expression correlates with increased levels of the tumour‐infiltrating lymphocyte (TIL) transcriptome signature.^79^ Immunohistochemical experiments further demonstrated higher levels of CD8 protein and significant upregulation of PDCD1 (which encodes the PD‐1 protein) in ARID1A‐depleted tumours.^64^ Gastrointestinal cancers with ARID1A mutations tend to exhibit elevated signatures of anti‐tumour immune cells, including CD8+ T cells, natural killer cells, activated Th1, activated dendritic cells and MHC class molecules, all of which have tumour‐suppressive effects.^61^ Interestingly, research has shown that innate immune cells like macrophages and neutrophils can infiltrate the liver and contribute to hepatocellular carcinoma development in the absence of ARID1A in mice. Furthermore, ARID1A has been found to interact with EZH2, a protein associated with immune regulation, and this interaction may impact T‐cell immunity.^83^, ^84^, ^85^ It's worth noting that the influence of ARID1A on immune cells can vary across different cancer types.

#### 2.4.3 Cytokines and inflammation

Cytokines are essential messengers that facilitate communication between immune and non‐immune cells. They are frequently released in the tumour microenvironment (TME) in close association with immune cells like lymphocytes and macrophages. One of these TME cytokines, interleukin‐6 (IL‐6), is mainly produced by T cells and macrophages and has a central role in creating an immunosuppressive TME. IL‐6 activates the signal transducer and transcriptional activator 3 (STAT3) through the JAK/STAT pathway, which, in turn, encourages inflammatory responses and the development of tumours.^86^ Interestingly, ARID1A deficiency has been linked to increased cytokine release in GC. Several studies have suggested that mutations in both ARID1A and PIK3CA synergistically promote tumour growth by causing sustained overproduction of IL‐6.^87^, ^88^ These studies found that the absence of ARID1A leads to the regulation of TILs, particularly CD8+ T lymphocytes, through IL‐6 within the TME. This supports the idea that tumours lacking ARID1A may be more susceptible to immunotherapy. Hepatic deficiency of ARID1A has also been associated with increased release of pro‐inflammatory cytokines, such as tumour necrosis factor‐alpha (TNF‐α) and IL‐6. This cytokine release can lead to steatohepatitis and liver tumorigenesis in mice, primarily through the activation of STAT3 and NF‐κB signalling pathways.^89^ In an inflammatory model, ARID1A deficiency resulted in elevated IL‐6 expression in mouse acinar cells due to epigenetic regulation of ARID1A‐related histone acetylation. Knockdown of ARID1A was shown to suppress the levels of Th1‐type chemokines (CXCL9 and CXCL10) and T cell effector molecules (IL‐2 and IFN‐γ) within the TME.^83^ These findings emphasize the complex interplay between ARID1A, cytokine release and the immune response in cancer.

#### 2.4.4 Tumour angiogenesis, invasion and metastasis

The processes of angiogenesis, invasion and metastasis are fundamental properties of tumour biology and are significant contributors to cancer‐associated mortality. These processes are often orchestrated within the context of an immunosuppressive TME. Epigenetic alterations play a key role in initiating tumour development and promoting invasion and metastasis. In the case of GC, inactivation of ARID1A has been shown to enhance invasion and metastasis. When the ARID1A gene is knocked out in most GC cell lines, it leads to increased cell proliferation, primarily through the upregulation of mRNA levels of key cell cycle regulators such as E2F1 and CCNE1. Conversely, the restoration of ARID1A inhibits the proliferation of these cancer cells.^90^ Deficiency of ARID1A is associated with larger tumour size, increased invasion depth, lymph node metastasis and a poor prognosis in gastric carcinomas that are EBV (−) and have preserved MLH1 expression.^57^ Immunohistological analyses have further supported these findings by showing a significant correlation between abnormal ARID1A expression and factors like lymphatic invasion and lymph node metastasis.^56^

Indeed, the impact of ARID1A inactivation extends beyond GC and is observed in various other types of cancer as well.^91^ In human endothelial cells, for instance, ARID1A inactivation through shRNA enhances cell proliferation and promotes migration and invasion, suggesting a broader role in angiogenesis and cellular mobility. Additionally, ARID1A loss has been linked to increased angiogenesis and the accelerated progression of HCC through Ang2‐dependent mechanisms.^92^ In breast cancer cells, ARID1A deficiency has been associated with enhanced migration, invasion and angiogenesis.^93^ Conversely, the activation of ARID1A has been shown to upregulate its downstream targets, leading to the suppression of HCC cell proliferation and migration. One of the mechanisms involves the inhibition of the lncRNA MVIH.^52^

## 3 POTENTIAL THERAPEUTIC TARGETS IN ARID1A MUTANT GC

**FIGURE 2:**
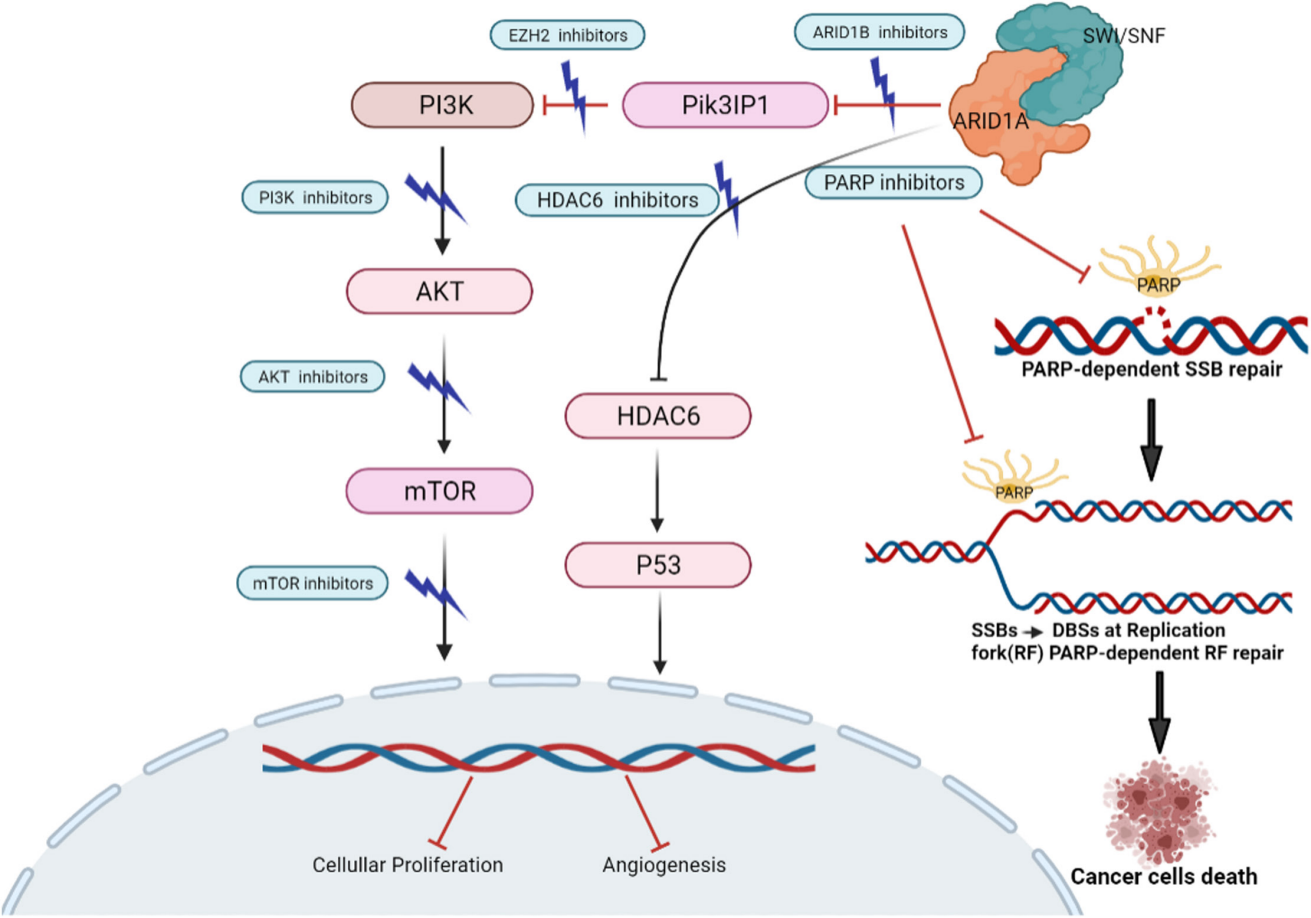
Therapeutic targets in AT‐rich interaction domain 1A (*ARID1A*)‐mutated cancers. Novel targeting of several key regulators in the phosphatidylinositol 3 kinase/AKT/mammalian target of rapamycin (Pl3AKT/mTOR) pathway and switch/sucrose non‐fermentable (SWI/SNF) complex modulate downstream effectors to inhibit cellular proliferation and promote apoptosis.

The absence of tumour suppressor factors such as ARID1A can create specific weaknesses in cancer cells that can be potentially targeted for treatment. Patients with ARID1A mutations in GC may be more responsive to immunotherapy, emphasizing the potential for combining different treatment strategies to improve the clinical outcomes of immune checkpoint inhibitors (ICI). Researchers have extensively studied targeted therapies for ARID1A‐mutated gastric cancer, including inhibitors of poly (ADP‐ribose) polymerase (PARP), PI3K/AKT, ATR, enhancer of EZH2 and histone deacetylase (HDAC).^81^, ^94^, ^95^, ^96^, ^97^ The combination of these targeted therapies with immune checkpoint inhibitors (ICIs) shows potential for producing synergistic effects in the treatment of cancers lacking ARID1A. Exploring the interactions and outcomes of combining immunotherapy with targeted therapy in ARID1A‐deficient cancers is essential for advancing treatment options and potentially enhancing patient outcomes. This approach has the potential to lead to more effective and personalized treatments for individuals with ARID1A‐mutated gastric cancer and other cancers with similar genetic changes (Figure 2).

### 3.1 PARP inhibitors

Defects in the DNA damage response can directly lead to genomic instability, which in turn contributes to the development of cancer. PARP is a crucial player in various cellular processes, including DNA repair, replication and transcription. When single‐strand breaks occur in DNA, PARP induces poly (ADP‐ribosylation) (PARylation) and regulates DNA repair by interacting with various proteins either directly or indirectly.^98^, ^99^ Inhibitors that target PARP directly have been found to block the repair of single‐strand breaks, leading to defects in homologous recombination and initiating synthetic lethal anti‐cancer strategies.^98^, ^100^ ARID1A has been shown to play a regulatory role in DNA damage checkpoints for double‐stranded breaks.^63^ Consequently, the loss of ARID1A sensitizes cancer cells to PARP inhibitors and inhibitors of DNA damage checkpoint kinase, both in laboratory studies and in animal models.^101^ While PARP inhibitors have shown promise, their effectiveness as monotherapy is limited, with progression‐free survival benefits typically lasting only a few months and no significant improvements in OS. Currently, combining PARP inhibitors with ICI is a promising approach. There is a significant overlap between PARP inhibitors and the mechanisms of tumour‐associated immunosuppression.^99^ Combination therapies, such as olaparib (a PARP inhibitor) and BKM120 (a PI3K inhibitor), have demonstrated potential in the treatment of GC.^102^

Data from preclinical and translational research on three different combinations of PARP inhibitors and ICI are promising. These combinations, which include olaparib/durvalumab, niraparib/pembrolizumab and BGB‐A317/BGB‐290, have all demonstrated some degree of antitumor activity.^103^ To thoroughly evaluate the safety and effectiveness of these combinations for treating ARID1A‐deficient tumours, it is crucial to conduct additional clinical trials. These trials will yield important insights into the potential of these therapies to enhance the outcomes of patients with ARID1A‐mutated cancers.

### 3.2 PI3K/AKT inhibitors

Comprehensive studies on the biological behaviour of GC have revealed that intracellular signals can activate the PI3K/AKT/mTOR pathway. This pathway plays a central role in cell physiology by transmitting signals in response to extracellular stimuli and regulates processes such as cell growth, the cell cycle, protein transcription and metabolism.^104^, ^105^ Abnormal activation of the PI3K/AKT/mTOR signalling pathway has been observed in GC tissues, and various molecular targeted inhibitors have been developed to counteract this pathway's dysregulation.^106^, ^107^, ^108^ However, many PI3K inhibitors have shown high cytotoxicity, and clinical results have been unsatisfactory thus far. There are a few PI3K inhibitors, such as idelalisib, copanlisib and duvelisib, that have received approval for cancer treatment.^109^ Furthermore, a phase I clinical trial (NCT03218826) involving AZD8186, a targeted PI3K inhibitor, is currently underway for various advanced solid tumours, including gastric cancer, and it commenced in March 2018. AZD8186 is anticipated to hold great promise in clinical applications because of its specific features and benefits in targeting the PI3K pathway.

Recent research has indicated that the loss of ARID1A can promote proliferation, migration and invasion in cancer cells by activating the AKT signalling pathway.^108^ Therefore, AKT inhibitors have the potential to be effective in ARID1A‐deficient tumours. MK2206 is the first allosteric AKT inhibitor with high selectivity that entered clinical trials.^110^ Phase I studies have shown that patients with GC tolerated chronic administration of MK2206 well. However, in a subsequent phase II clinical trial involving 70 patients with advanced GC, the OS was 5.1 months, and the efficacy endpoint was less than 6.5 months, suggesting significant side effects. AZD5363 inhibitors have demonstrated good safety and tolerability in phase I clinical studies involving advanced solid tumours conducted in Japan, with common adverse reactions being diarrhoea, hyperglycemia and nausea.^111^ Currently, this drug is being examined in a phase II clinical study for advanced GC (NCT02451956), and it holds promise for future applications.

Inhibitors of mTOR have been approved for the treatment of various cancers. In certain cancer types, such as those with loss‐of‐function mutations in mTOR genes like TSC1, TSC2 or STK11, mTOR inhibitors have shown sensitivity.^110^ Moreover, PI3K/AKT/mTOR inhibitors, when combined with other therapies, have demonstrated effectiveness against ARID1A‐deficient cancers.^111^ One example is NVP‐BEZ235, a novel PI3K/mTOR dual inhibitor that targets the protein kinase domain, inhibiting both the catalytic subunit (P110A) of PI3K and mTOR activity.^43^ Studies have indicated that NVP‐BEZ235 can significantly reduce GC cell motility and promote apoptosis, particularly in cells with PI3KCA mutations.^41^ Therefore, a deeper understanding of the immunomodulatory effects of PI3K/AKT/mTOR inhibitors may pave the way for their efficient combination with other therapeutic agents, including immune checkpoint blockers, to enhance clinical outcomes for patients with ARID1A‐deficient cancers.

### 3.3 EZH2 inhibitors

EZH2 is a gene responsible for encoding a histone‐lysine N‐methyltransferase, which plays a role in DNA methylation. Its function involves inhibiting the transcription of downstream genes. The methylation activity of EZH2 encourages the formation of heterochromatin and the silencing of genes. Mutations and overexpression of EZH2 have been linked to various cancer types, including breast cancer, prostate cancer, melanoma and bladder cancer. When EZH2 is abnormally activated, it leads to the suppression of tumour suppressor genes. Conversely, inhibiting EZH2's activity can slow down tumour growth. Genetic and post‐transcriptional abnormalities in EZH2 are frequently observed in many cancer types, and EZH2 targets involved in chromatin remodelling are often used to develop strategies based on synthetic lethality.^86^ Bitler et al.^95^ reported that inhibiting EZH2 can effectively induce apoptosis in ARID1A mutant cells. Additionally, treatment with the EZH2 inhibitor GSK126 resulted in the regression of ARID1A mutant tumours. This suggests that targeting EZH2 could be a potential therapeutic strategy for ARID1A‐deficient cancers.

The study by Wu and colleagues demonstrated that a combination of EZH2 inhibitors, ABT263 and GSK126, was effective in inhibiting the growth of established tumours in a mouse model of ovarian clear cell carcinoma with mutations in ARID1A and PIK3CAH1047R.^112^ This research supports the idea that targeting the methyltransferase activity of EZH2 in ARID1A mutant cells with specific EZH2 inhibitors could be a promising synthetic lethal therapy strategy. Furthermore, recent experiments have shown that EZH2, along with the PRC2 complex, inhibits the production of T‐helper 1 chemokines CXCL9 and CXCL10 in ovarian and colon cancer. This inhibition affects the recruitment of effector T cells to the tumour microenvironment.^85^ This finding highlights the potential immunomodulatory effects of targeting EZH2 in cancer therapy. However, it's important to note that there have been limited preclinical or clinical studies on the efficacy of EZH2 inhibitors combined with ICI specifically for ARID1A mutant GC. More research is needed to explore the potential benefits of this combination therapy in the context of ARID1A‐deficient cancers, including GC.

### 3.4 HDAC6 inhibitors

Epigenetic drivers play a crucial role in the regulation of gene expression, and their dysregulation is often associated with cancer. Targeting these epigenetic modifications presents a promising avenue for cancer therapy. Epigenetic therapy can potentially uncover tumour‐associated antigens that are silenced by epigenetic modifications, making them visible to the immune system.^113^ The reversibility of epigenetic changes further supports their potential as therapeutic targets. There are two main classes of epigenetic drugs that have been developed: DNMTs and histone deacetylase inhibitors (HDACIs).^114^ In the context of GC, studies have shown that increased expression of HDACs in GC cells is associated with cancer staging, potential for distant metastasis and decreased OS in metastatic lymph nodes. This suggests that HDACIs could be a promising therapeutic approach for GC. One example is trichostatin A, an HDACI that has demonstrated inhibitory effects on GC cell proliferation.^38^ Additionally, HDAC6, a histone deacetylase, appears to be a transcriptional target of the SWI/SNF complex, and when ARID1A is inactivated, HDAC6 is de‐inhibited. In this context, the HDAC6 inhibitor ACY1215 has shown promise in exerting antitumor effects, both as a monotherapy and in combination with other drugs, in various cancer types, including GC.^39^ These findings suggest that epigenetic therapies, particularly HDACIs, have the potential to be effective in the treatment of GC, especially in cases involving ARID1A mutations. Further research and clinical trials are needed to evaluate the safety and efficacy of these treatments in GC patients.^40^, ^42^, ^43^, ^44^

### 3.5 Other targeted therapeutic measures

Several targeted agents have shown promise in the context of ARID1A‐deficient cancers, including inhibitors of YES1, cell cycle modulators and ARID1B. These agents have the potential to exploit synthetic lethal interactions to selectively target ARID1A‐mutant cancer cells.^42^ Here are some key findings related to these targeted agents: (1) Inhibition of YES1: High‐throughput drug screening experiments in clear cell ovarian carcinoma revealed a synthetic lethal relationship between ARID1A mutations and the kinase inhibitor dasatinib, which targets YES1. ARID1A‐deficient cells were found to be sensitive to dasatinib treatment, leading to G1‐S cell cycle arrest, and this effect was dependent on the presence of P21 and Rb.^44^ (2) Cell cycle modulators: Another study developed a screening method to identify drugs that selectively regulate the cell cycle in ARID1A‐deficient cells. This approach aims to identify compounds that enhance the susceptibility of ARID1A‐deficient cells to treatment by modulating cell cycle progression. While this screening method has shown promise, further validation in both in vitro and in vivo settings is necessary to confirm the effectiveness of the target compounds. (3) ARID1B: ARID1B is a homologous protein to ARID1A and is a component of the SWI/SNF chromatin remodelling complex. While ARID1A mutations are well‐studied, the role of ARID1B in cancer and its potential as a therapeutic target in ARID1A‐deficient cancers are areas of ongoing research. It's important to note that the application of these targeted agents has not been extensively studied in the context of ARID1A‐mutant GC. Further clinical trials and research are needed to explore their potential as therapeutic options for this specific cancer subtype. The findings in other cancer types, such as clear cell ovarian carcinoma, may provide valuable insights into potential treatment strategies for ARID1A‐deficient GC.

## 4 CONCLUSION

Immunotherapy for GC has gained significant attention recently, especially immune checkpoint inhibitor (ICI)‐based immunotherapy, which shows promise in managing GC comprehensively. However, successful immunotherapy relies on identifying and evaluating specific patient populations as potential biomarkers. Currently, relying on single biomarkers for GC falls short in accurately predicting which patients will benefit most from immunotherapy.

This review underscores the potential of ARID1A in immunotherapy for GC. ARID1A contributes to maintaining genome stability, influencing DNA methylation and shaping the TME. Recent advances in cancer genomics have established ARID1A's role as a crucial tumour suppressor, functioning as both a gatekeeper and caretaker. Consequently, ARID1A mutations have emerged as promising targets and potential biomarkers for immunotherapy in GC. However, further research is needed to delve into the causal relationships and mechanisms linking ARID1A with DNA MMR. Ongoing investigations are actively exploring how ARID1A operates in tumour suppression and its intricate interactions with other oncogenic networks.

It's important to note that the loss or mutation of ARID1A alone may not be sufficient for accurately predicting the effectiveness of immunotherapy in all cases of GC. The clinical relevance of PD‐L1 in advanced GC is contingent on factors such as ARID1A mutation and ATM expression.^45^ For instance, research by Goswami et al.^46^ has demonstrated that when combined with CXCL13 expression, ARID1A mutation can serve as part of a composite biomarker that predicts the response to immune checkpoint therapy in metastatic uroepithelial carcinoma. In the context of GC using ARID1A in conjunction with other biomarkers offers a more effective approach for forecasting the outcomes of immunotherapy. Future studies that leverage transcriptomics, immunomics and artificial intelligence are poised to provide comprehensive insights into how ARID1A deficiency relates to genome instability and the remodelling of the TME. By combining effective biomarkers, we may establish predictive or prognostic factors for ICI‐based therapy in AGC.

## AUTHOR CONTRIBUTIONS

**Xuemei Zhang:** Data curation (equal); investigation (equal); validation (equal); writing – original draft (equal). **Youzhi Zhang:** Investigation (equal); project administration (equal); writing – original draft (equal). **Qiaoyun Zhang:** Methodology (equal); validation (equal). **Mengyao Lu:** Investigation (equal); validation (equal). **Yuan Chen:** Formal analysis (equal); methodology (equal). **Xiaoyu Zhang:** Conceptualization (equal); validation (equal); writing – original draft (equal). **Peng Zhang:** Conceptualization (equal); investigation (equal); methodology (equal); supervision (equal); writing – original draft (equal).

## FUNDING INFORMATION

This work was funded by the National Natural Science Foundation of China (No. 81974483 and 82072589), the Key Project of the Education Commission of Hubei Province (D20202802) and Chinese Society of Clinical Oncology (CSCO)‐Hengrui Cancer Research Fund (Y‐HR2020QN‐0946).

## CONFLICT OF INTEREST STATEMENT

This research was conducted in the absence of any commercial or financial relationships that could be construed as a potential conflict of interest.

## Data Availability

All data have been included within this manuscript.
